# Histone Deacetylase Inhibitor Valproic Acid Promotes the Differentiation of Human Induced Pluripotent Stem Cells into Hepatocyte-Like Cells

**DOI:** 10.1371/journal.pone.0104010

**Published:** 2014-08-01

**Authors:** Yuki Kondo, Takahiro Iwao, Sachimi Yoshihashi, Kayo Mimori, Ruri Ogihara, Kiyoshi Nagata, Kouichi Kurose, Masayoshi Saito, Takuro Niwa, Takayoshi Suzuki, Naoki Miyata, Shigeru Ohmori, Katsunori Nakamura, Tamihide Matsunaga

**Affiliations:** 1 Department of Clinical Pharmacy, Graduate School of Pharmaceutical Sciences, Nagoya City University, Nagoya, Japan; 2 Educational Research Center for Clinical Pharmacy, Faculty of Pharmaceutical Sciences, Nagoya City University, Nagoya, Japan; 3 Department of Environmental and Health Science, Tohoku Pharmaceutical University, Sendai, Japan; 4 Department of Food Science and Technology, Graduate School of Marine Science and Technology, Tokyo University of Marine Science and Technology, Tokyo, Japan; 5 DMPK Research Laboratory, Mitsubishi Tanabe Pharma Corporation, Toda, Japan; 6 Graduate School of Medical Science, Kyoto Prefectural University of Medicine, Kyoto, Japan; 7 Department of Organic and Medicinal Chemistry, Graduate School of Pharmaceutical Sciences, Nagoya City University, Nagoya, Japan; 8 Department of Biochemical Pharmacology and Toxicology, Shinshu University, Matsumoto, Japan; University of Kansas Medical Center, United States of America

## Abstract

In this study, we aimed to elucidate the effects and mechanism of action of valproic acid on hepatic differentiation from human induced pluripotent stem cell-derived hepatic progenitor cells. Human induced pluripotent stem cells were differentiated into endodermal cells in the presence of activin A and then into hepatic progenitor cells using dimethyl sulfoxide. Hepatic progenitor cells were matured in the presence of hepatocyte growth factor, oncostatin M, and dexamethasone with valproic acid that was added during the maturation process. After 25 days of differentiation, cells expressed hepatic marker genes and drug-metabolizing enzymes and exhibited drug-metabolizing enzyme activities. These expression levels and activities were increased by treatment with valproic acid, the timing and duration of which were important parameters to promote differentiation from human induced pluripotent stem cell-derived hepatic progenitor cells into hepatocytes. Valproic acid inhibited histone deacetylase activity during differentiation of human induced pluripotent stem cells, and other histone deacetylase inhibitors also enhanced differentiation into hepatocytes. In conclusion, histone deacetylase inhibitors such as valproic acid can be used to promote hepatic differentiation from human induced pluripotent stem cell-derived hepatic progenitor cells.

## Introduction

Induced pluripotent stem (iPS) cells, originally generated from human fibroblasts, are pluripotent and have infinite proliferative potential *in vitro* [1]. Human iPS (hiPS) cells are expected to have various applications, including for studying hepatic drug metabolism and toxicity [2]. Furthermore, hiPS cell-derived hepatocytes may constitute a source of cells for transplantation in the case of a severe liver disease. Previous studies reported hepatic differentiation from hiPS cells using many cytokines such as recombinant growth factors [3]–[6]. However, cytokines require careful handling because of their structural instability; moreover, they have insufficient hepatic-differentiation activities. In contrast, hepatic differentiation methods that use a combination of cytokines and overexpression of transcriptional factors by viral vectors or co-culture with other cells have been reported [7]–[10]. Although these methods enhance hepatic functions in differentiated cells, they require considerable technical skills and specialized materials such as modified adenoviruses. Large-scale production of hiPS cell-derived hepatocytes is needed for drug development studies and cell transplantation. However, it is difficult to prepare the reagents required for differentiation of these cells in sufficient quantities using the methods reported previously, and product validation is required.

In general, small-molecule compounds can be synthesized with a high quantity, stability, and purity, and can be used with low risk and lot-to-lot variations; they will thus be highly useful for differentiation into hepatocytes from hiPS cells as differentiation-inducing-factors. Several studies have reported the use of small-molecule compounds in hepatic differentiation from embryonic stem (ES)/iPS cells [11]–[13]. However, simple and inexpensive methods for highly efficient hepatic differentiation from hiPS cells have not been established.

Valproic acid (VPA) is a branched short-chain fatty acid that is widely used as an antiepileptic, antimanic, and antimigraine prophylactic drug. The drug raises γ-aminobutyric acid (GABA) concentrations in the human brain by inhibiting GABA transaminase, which participates in GABA degradation, thereby controlling epilepsy [14]. VPA is also known as a potent histone deacetylase (HDAC) inhibitor that is currently being evaluated for cancer treatment [15]. In previous stem cell studies, VPA enabled efficient induction of pluripotent stem cells without introduction of the c-Myc oncogene [16].

Hay *et al*. studied the early stages of hepatic differentiation of human ES cells using sodium butyrate (NaB) [17], which is an HDAC inhibitor [18]. VPA was also used to promote hepatic differentiation from human bone marrow stromal stem cells [19] and mouse ES cells [20]. In both studies, differentiation was initiated by treating undifferentiated cells with VPA, instead of activin A, which is commonly used to differentiate ES/iPS cells into endodermal cells. Although these compounds were used in the initial differentiation processes of undifferentiated cells, the effects of VPA on the maturation processes during differentiation from hiPS cells into hepatocytes remain unknown. Moreover, HDAC inhibitors other than VPA or NaB have not been examined. The present study tested the potential of VPA as a small-molecule compound for hepatic differentiation from hiPS cells. The effect of VPA was evaluated using a multidisciplinary approach that included the assessment of hepatic marker gene expression and enzyme activity, as well as an immunofluorescence assay. The mechanism underlying the VPA-mediated effects was identified using selective inhibitors of HDAC, GABA transaminase, and ion channels, and it was confirmed by functional assays.

## Materials and Methods

### Materials

The hiPS cell lines Windy [21], Dotcom [7], and Fetch [6], derived from the human embryonic lung fibroblast cell line MRC-5, were provided by Umezawa *et al*. of the National Center for Child Health and Development. Cryopreserved human primary hepatocytes (HPHs; lot. HPCH10/0910463; 10 donors aged 32–76 years) were obtained from XenoTech (Lenexa, KS). Activin A and the hepatocyte growth factor were purchased from PeproTech Inc. (Rocky Hill, NJ). Fetal bovine serum was purchased from Biowest (Nuaillé, France). Accutase was purchased from MS TechnoSystems (Osaka, Japan). Minimal essential medium nonessential amino acids, oncostatin M, dexamethasone, Y-27632, rifampicin (RIF), VPA, gabaculine, trichostatin A (TSA), NaB, vorinostat, procainamide, lidocaine, zonisamide, nifedipine, and midazolam were purchased from Wako Pure Chemical Industries (Osaka, Japan). BD Matrigel matrix Growth Factor Reduced, bupropion, hydroxybupropion, 4′-hydroxydiclofenac-^13^C_6_, and hydroxybupropion-d_6_ were purchased from BD Biosciences (Bedford, MA). Dimethyl sulfoxide, 2-mercaptoethanol, vigabatrin, ethosuximide, MS-275, acetaminophen, 7-hydroxycoumarin glucuronide, 7-hydroxycoumarin sulfate, and caffeine were purchased from Sigma-Aldrich Co. (St. Louis, MO). (±)-Bufuralol hydrochloride, 1′-hydroxymidazolam, acetaminophen-d_4_, 4′-hydroxymephenytoin-d_3_, and 1′-hydroxybufuralol-d_9_ were purchased from Toronto Research Chemicals (North York, ON, Canada). Diclofenac was purchased from Ultrafine (Manchester, UK). (*S*)-Mephenytoin was purchased from Enzo Life Sciences (Farmingdale, NY). Phenacetin and 7-hydroxycoumarin were purchased from Nacalai Tesque (Kyoto, Japan). 4′-Hydroxymephenytoin, 1′-hydroxybufuralol, and 4′-hydroxydiclofenac were purchased from Sumika Chemical Analysis Service, Ltd. (Tokyo, Japan). 1′-Hydroxymidazolam-d_4_ was purchased from Cerilliant Corporation (Round Rock, TX). The mouse monoclonal antihuman albumin (ALB) antibody was purchased from Abcam (Cambridge, UK). KnockOut Serum Replacement (KSR), KnockOut Dulbecco's modified Eagle medium, Roswell Park Memorial Institute (RPMI) + GlutaMax medium, GlutaMax, and Alexa Fluor 568 goat antimouse IgG were purchased from Invitrogen Life Technologies Co. (Carlsbad, CA). Cosmedium 004 (Cosmedium) was purchased from COSMO BIO Co. (Tokyo, Japan). T247 and NCC149 were synthesized as reported previously [22], [23]. All other reagents were of the highest quality available.

### Differentiation of hiPS cells into hepatocytes

**Figure 1:**
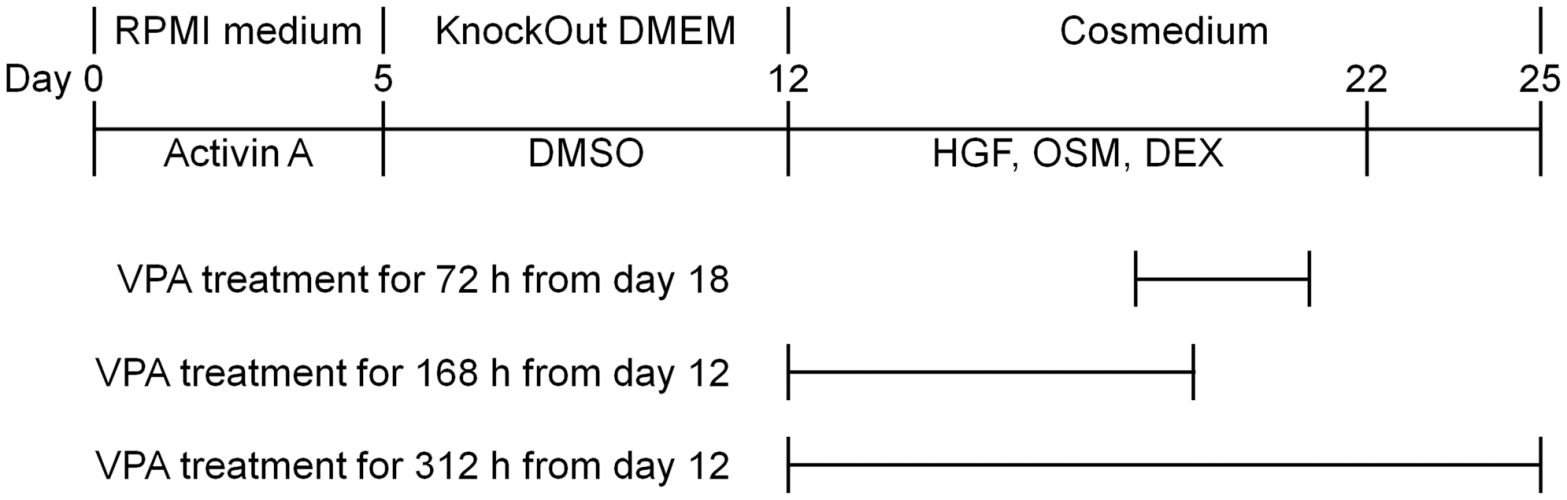
Schematic protocol for hepatic differentiation of hiPS cells. Human iPS (hiPS) cells were differentiated into endodermal cells using Roswell Park Memorial Institute (RPMI) + GlutaMAX medium containing 100 ng/mL of activin A for 5 days, and then into hepatic progenitor cells (HPCs) using KnockOut Dulbecco's modified Eagle medium (KnockOut DMEM) containing 1% dimethyl sulfoxide (DMSO) for 7 days. Thereafter, HPCs were matured using Cosmedium 004 (Cosmedium) containing 10 ng/mL of hepatocyte growth factor (HGF), 20 ng/mL of oncostatin M (OSM), and 100 nM of dexamethasone (DEX) for 10 days. Finally, the cells were cultured in only Cosmedium for 3 days. Valproic acid (VPA) was added to the culture medium for 72 h from day 18, 168 h from day 12, or 312 h from day 12 at a final concentration of 2 mM.

Undifferentiated hiPS cells were cultured as reported previously [6]. The hiPS cells were differentiated into endodermal cells by culturing in RPMI + GlutaMax medium containing 0.5% fetal bovine serum and 100 ng/mL of activin A for 3 days, followed by culturing in RPMI + GlutaMax medium containing 2% KSR and 100 ng/mL of activin A for 2 days. After induction of differentiation, the endodermal cells were dissociated using Accutase for 5 min at 37°C and passaged onto 24- or 96-well plates coated with a thin layer of BD Matrigel matrix Growth Factor Reduced. Y-27632 was added to the culture medium for 24 h after passage at a final concentration of 10 µM. The endodermal cells were differentiated into hepatic progenitor cells (HPCs) by culturing in KnockOut Dulbecco's modified Eagle medium containing 20% KSR, 1% GlutaMax, 1% minimal essential medium nonessential amino acids, 0.1-mM 2-mercaptoethanol, and 1% dimethyl sulfoxide for 7 days. HPCs were then matured by culturing in Cosmedium containing 10 ng/mL of hepatocyte growth factor, 20 ng/mL of oncostatin M, and 100-nM dexamethasone for 10 days. Finally, the cells were cultured in Cosmedium for 3 days. VPA was added to the culture medium for 72 h from day 18 (72-h VPA treatment), for 168 h from day 12 (168-h VPA treatment), or for 312 h from day 12 (312-h VPA treatment) at a final concentration of 2 mM (Fig. 1). Other compounds (1-µM TSA, 5-mM NaB, 5-µM vorinostat, 1.2-µM T247, 1-µM MS-275, 1-µM NCC149, 100-µM gabaculine, 100-µM vigabatrin, 25-µM procainamide, 1-mM lidocaine, 500-µM ethosuximide, 1-µM nifedipine, and 30-µM zonisamide) were added to the culture medium for 168 h from day 12. In the induction study, differentiated cells were treated with 40-µM RIF for the final 48 h of culture.

### Cryopreserved human hepatocyte cultures

Cryopreserved HPHs were thawed using a thawing medium without additives (Biopredic, Rennes, France) and seeded on collagen I-coated plates in basal hepatic cell medium (Biopredic) containing additives for hepatocyte seeding medium (Biopredic) for 12 h at 37°C. The medium was then changed to the basal hepatic cell medium containing additives for hepatocyte culture (Biopredic), and the cells were cultured for 36 h.

### Real-time RT-PCR analysis

Total RNA was extracted using an RNeasy Mini Kit (QIAGEN, Valencia, CA) and first-strand cDNA was generated using a PrimeScript RT Reagent Kit (Takara Bio Inc., Otsu, Japan), according to the manufacturer's instructions. Real-time RT-PCR was performed using SYBR Premix EX Taq II (Takara Bio Inc.), and products were detected using an ABI 7300 Real Time PCR System (Applied Biosystems, Carlsbad, CA). The mRNA expression levels were normalized to those of glyceraldehyde-3-phosphate dehydrogenase. The primers used in this experiment are listed in Table 1.

**Table 1:** Sequences of primers for real-time RT-PCR analysis.

Gene names	Forward primer sequences (5′- -3′)	Reverse primer sequences (5′- -3′)
ALB	GAGCTTTTTGAGCAGCTTGG	GGTTCAGGACCACGGATAGA
AFP	AGCTTGGTGGTGGATGAAAC	TCTGCAATGACAGCCTCAAG
TAT	ATCTCTGTTATGGGGCGTTG	TGATGACCACTCGGATGAAA
PXR	AGGATGGCAGTGTCTGGAAC	AGGGAGATCTGGTCCTCGAT
CYP2C9	GACATGAACAACCCTCAGGACTTT	TGCTTGTCGTCTCTGTCCCA
CYP2C19	GAACACCAAGAATCGATGGACA	TCAGCAGGAGAAGGAGAGCATA
CYP3A4	CTGTGTGTTTCCAAGAGAAGTTAC	TGCATCAATTTCCTCCTGCAG
UGT1A1	CAGCAGAGGGGACATGAAAT	ACGCTGCAGGAAAGAATCAT
GAPDH	GAGTCAACGGATTTGGTCGT	GACAAGCTTCCCGTTCTCAG

The abbreviations used are: ALB, albumin; AFP, α-fetoprotein; TAT, tyrosine aminotransferase; PXR, pregnane X receptor; CYP, cytochrome P450; UGT, UDP-glucuronosyltransferase; GAPDH, glyceraldehyde-3-phosphate dehydrogenase.

### Immunofluorescence staining

Cells were fixed for 20 min at room temperature in 4% paraformaldehyde, and then permeabilized in methanol for 5 min at −30°C. After blocking with 2% skim milk for 30 min at room temperature, cells were incubated with the mouse monoclonal antihuman ALB antibody (dilution, 1∶200) for 60 min at room temperature, followed by incubation with a 1∶500 dilution of Alexa Fluor 568 goat antimouse IgG for 60 min at room temperature. Finally, cells were incubated with 1 µg/mL of 4′,6-diamidino-2-phenylindole for 5 min at room temperature and observed under an ECLIPSE N*i* microscope (NIKON Inc., Tokyo, Japan).

### Determination of drug-metabolizing enzyme activities

Differentiated hiPS cells were incubated in Cosmedium containing 40-µM phenacetin, 50-µM bupropion, 5-µM diclofenac, 100-µM (*S*)-mephenytoin, 5 µM-bufuralol, 5-µM midazolam, and 10-µM 7-hydroxycoumarin for 6 or 24 h at 37°C. Thereafter, 40-µL aliquots of reaction medium were collected, and the reactions were stopped by adding 40 µL of ice-cold acetonitrile containing stable isotope-labeled internal standards for quantification. The metabolites were measured using ultra performance liquid chromatography–tandem mass spectrometry (UPLC–MS/MS). The probe substrates of drug-metabolizing enzymes and the metabolites used in this experiment are summarized in Table 2.

**Table 2:** Probe substrates of drug-metabolizing enzyme and the metabolites.

Drug-metabolizing enzyme	Substrate	Metabolite
CYP1A1/2	Phenacetin	Acetaminophen
CYP2B6	Bupuropion	Hydroxybupropion
CYP2C9	Diclofenac	4′-Hydroxydiclofenac
CYP2C19	(*S*)-Mepehnytoin	4′-Hydroxymephenytoin
CYP2D6	Bufuralol	1′-Hydroxybufuralol
CYP3A4/5	Midazolam	1′-Hydroxymidazolam
UGT	7-Hydroxycoumarin	7-Hydroxycoumarin glucuronide
SULT	7-Hydroxycoumarin	7-Hydroxycoumarin sulfate

The abbreviations used are: CYP, cytochrome P450; UGT, UDP-glucuronosyltransferase; SULT, sulfotransferase.

For UPLC–MS/MS analyses, samples were thawed and centrifuged at 13,000 rpm for 3 min at 4°C. To measure metabolites, 2-µL aliquots of supernatant were injected into the UPLC–MS/MS apparatus. UPLC was performed using a Water ACQUITY UPLC system (Waters Corporation, Milford, MA). UPLC was performed using an Aquity UPLC BEH C18 column (2.1×50 mm, 1.7 µm), mobile phases of water containing 0.025% formic acid, and methanol containing 0.025% formic acid, at a flow rate of 0.8 mL/min, a column temperature of 55°C, and a sample temperature of 8°C. Eluents were analyzed in the multiple reaction monitoring mode, under positive and negative electrospray ionization conditions using a Waters Xevo TQ-S mass spectrometer. MS was performed using a capillary voltage of 0.5 kV, a source temperature of 150°C, a desolvation temperature of 650°C, a cone gas flow of 150 L/h, a desolvation gas flow of 1,200 L/h, and a collision gas flow of 0.18 mL/min using argon as the collision gas.

To correct for drug-metabolizing enzyme activities, plated cells were lysed and total protein content was measured using a Pierce BCA protein assay kit (Thermo Fisher Scientific Inc., Waltham, MA), according to the manufacturer's instructions.

### Determination of HDAC activity

Cells were incubated in 100 µL of Williams' medium E (without phenol red) with or without 2-mM VPA for 30 min at 37°C. Subsequently, 100 µL of HDAC-Glo I/II Reagent was added to each well, and the cells were incubated for 15 min at room temperature. After incubation, 100 µL of supernatant was transferred to white-walled plates, and the degrees of luminescence for 0.5 sec of integration time were measured using GloMAX-Multi+ (Promega). To correct for HDAC activity, cell numbers were counted using a Cell Counting Kit-8 (DOJINDO, Kumamoto, Japan) before assay of HDAC activity, according to the manufacturer's instructions.

### Statistical analysis

Levels of statistical significance were assessed using Student's *t*-test, and multiple comparisons were performed using analysis of variance followed by Dunnett's test. A *p* value of <0.05 was considered statistically significant.

## Results

### VPA-induced differentiation from hiPS cells into hepatocytes

**Figure 2:**
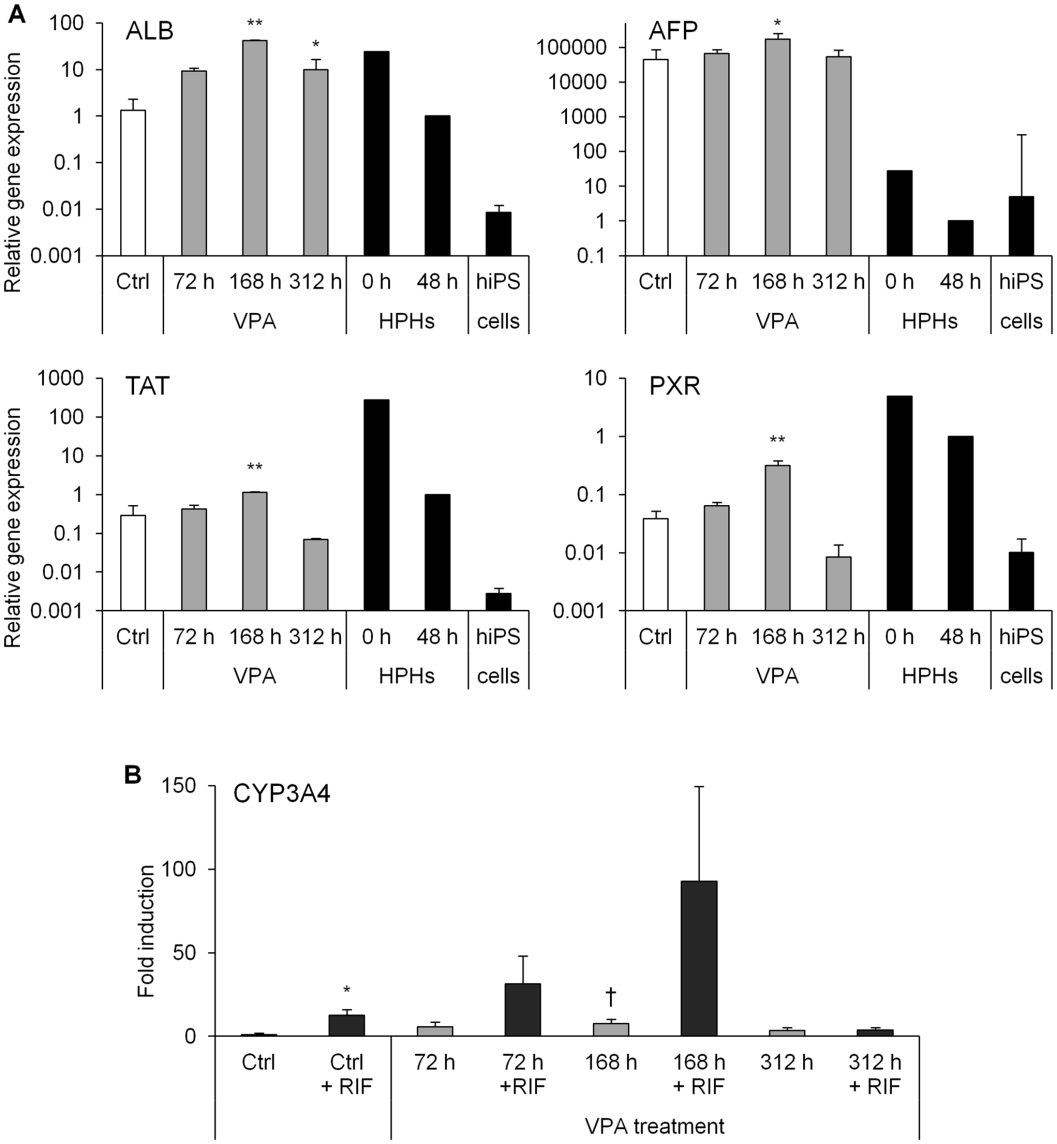
Effects of VPA on hepatic marker gene expression and induction of the CYP3A4 mRNA by RIF. Human iPS (hiPS) cells (Windy) were differentiated into hepatocytes. Valproic acid (VPA) was added to the medium for 72 h from day 18 (72 h), 168 h from day 12 (168 h), or 312 h from day 12 (312 h). (A) Cryopreserved human primary hepatocytes (HPHs) were cultured for 0 (just after thawing) and 48 h. Each bar represents the mean ± standard deviation (*n* = 3). The graph represents gene expression relative to that detected in HPHs cultured for 48 h. Levels of statistical significance compared with VPA-untreated hepatocyte-like cells [control (Ctrl)]: *P<0.05 and **P<0.01; and (B) hiPS cell-derived hepatocyte-like cells were treated with 40-µM rifampicin (RIF) for the last 48 h of culture. Each bar represents the mean ± standard deviation (*n* = 2–3). The graph represents gene expression relative to that detected in VPA-untreated hepatocyte-like cells without RIF. Levels of statistical significance compared with Ctrl in the RIF-untreated group (†), and RIF-treated group compared with each RIF-untreated group (*), respectively: †*P*<0.05 and *P<0.05. The abbreviations used are: AFP, α-fetoprotein; ALB, albumin; TAT, tyrosine aminotransferase; PXR, pregnane X receptor; CYP, cytochrome P450.

To investigate whether VPA promotes differentiation from hiPS cells into hepatocytes, the effects of VPA were examined at several time points. Hepatic differentiation from hiPS cells was evaluated by measuring the expression of ALB, α-fetoprotein (AFP), and tyrosine aminotransferase (TAT), which are hepatocyte-specific marker proteins, and of the pregnane X receptor (PXR), which is a nuclear receptor that regulates cytochrome P450 (CYP) 3A4 expression. Compared with the control group (VPA nontreatment), ALB and PXR mRNAs increased by 7- and 1.7-fold after the 72-h VPA treatment, respectively. These mRNAs also increased by 32- and 5-fold after the 168-h VPA treatment, respectively (Fig. 2A). After the 312-h VPA treatment, the ALB mRNA increased by 8-fold, whereas the PXR mRNA decreased to 0.2-fold. The TAT mRNA expression increased by 1.5- and 4-fold after 72- and 168-h VPA treatments, respectively, but it decreased to 0.3-fold after the 312-h VPA treatment. The AFP mRNA expression was altered by 1.5-, 3.8-, and 1.2-fold after the 72-, 168-h, and 312-h VPA treatments, respectively. HPHs were used to evaluate hepatic differentiation from hiPS cells. In drug-development studies, HPHs are usually tested after cultivation for a few days, whereas it is known that the function of HPHs is reduced dramatically by cultivation after thawing. Therefore, we used HPHs cultured for 48 h (HPHs 48 h) as the positive control. The mRNA expression of ALB in differentiated cells after the 168-h VPA treatment was 42-fold higher than that detected in HPHs 48 h, and the mRNAs of TAT and PXR were expressed at levels that were similar to those of HPHs 48 h. The AFP mRNA in all groups of differentiated cells was higher than that observed in HPHs 48 h.

CYP3A4, a major CYP isoform in the human liver, is also an excellent marker of hepatic differentiation. The mRNA expression of CYP3A4 in the differentiated cells increased by 5.7-, 7.5-, and 3.5-fold after the 72-, 168-, and 312-h VPA treatments, respectively, compared with control group (Fig. 2B). Furthermore, the CYP3A4 mRNA was markedly induced by treatment with RIF in the 72- and 168-h VPA treatment groups. However, the CYP3A4 mRNA expression was unaffected by RIF after the 312-h VPA treatment.

### Morphological changes and immunofluorescence staining of ALB

**Figure 3:**
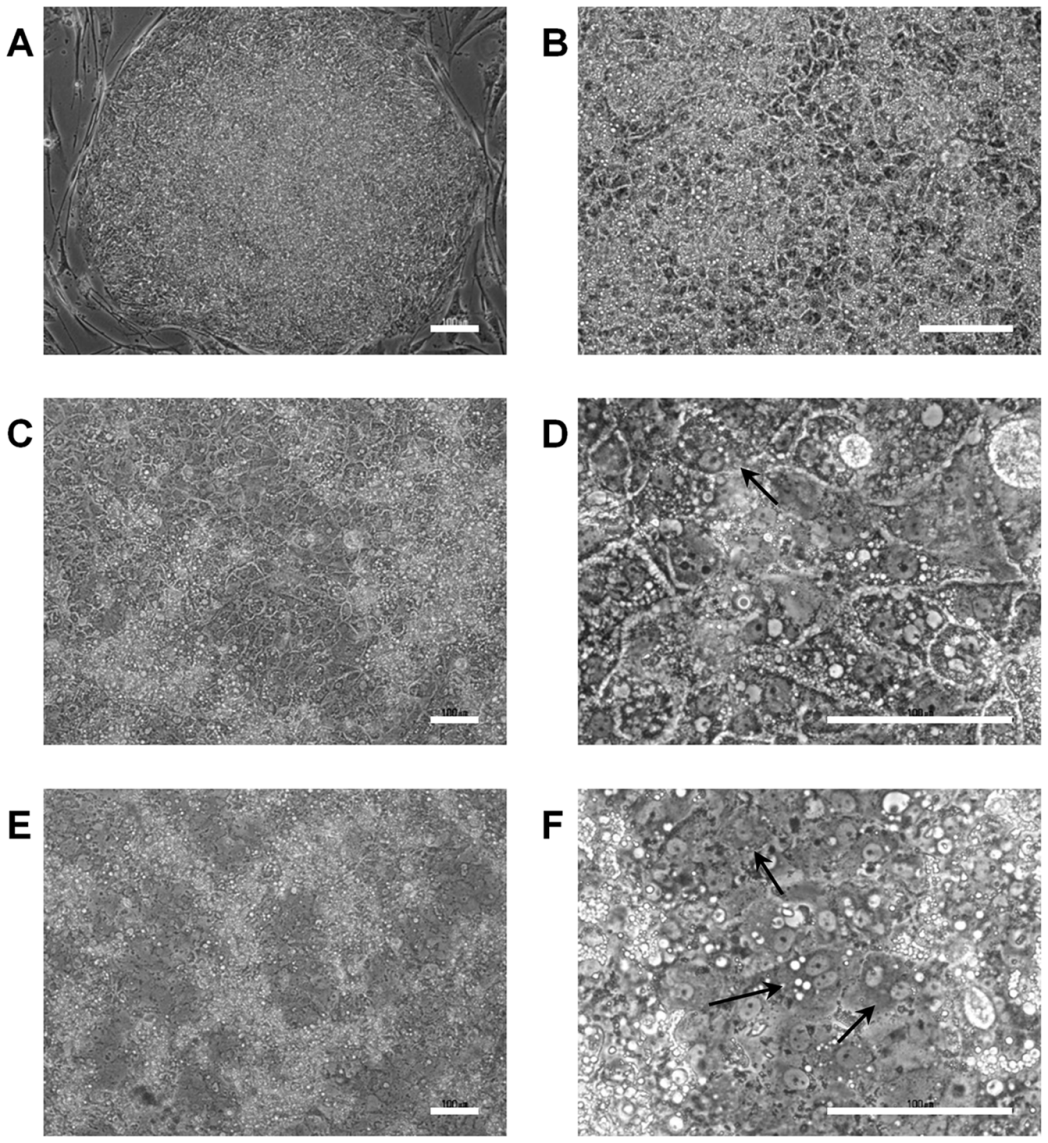
Morphological changes observed in hiPS cells during hepatic differentiation. The images show morphological changes in human iPS (hiPS) cells (Windy). (A) Undifferentiated hiPS cells; (B) hepatic progenitor cell-like cells after 12 days of differentiation; (C, D) hepatocyte-like cells after 25 days of differentiation in the absence of valproic acid (VPA); and (E, F) hepatocyte-like cells after 25 days of differentiation in the presence of 2-mM VPA for 168 h from day 12. Arrows indicate binuclear cells. Scale bar, 100 µm.

**Figure 4:**
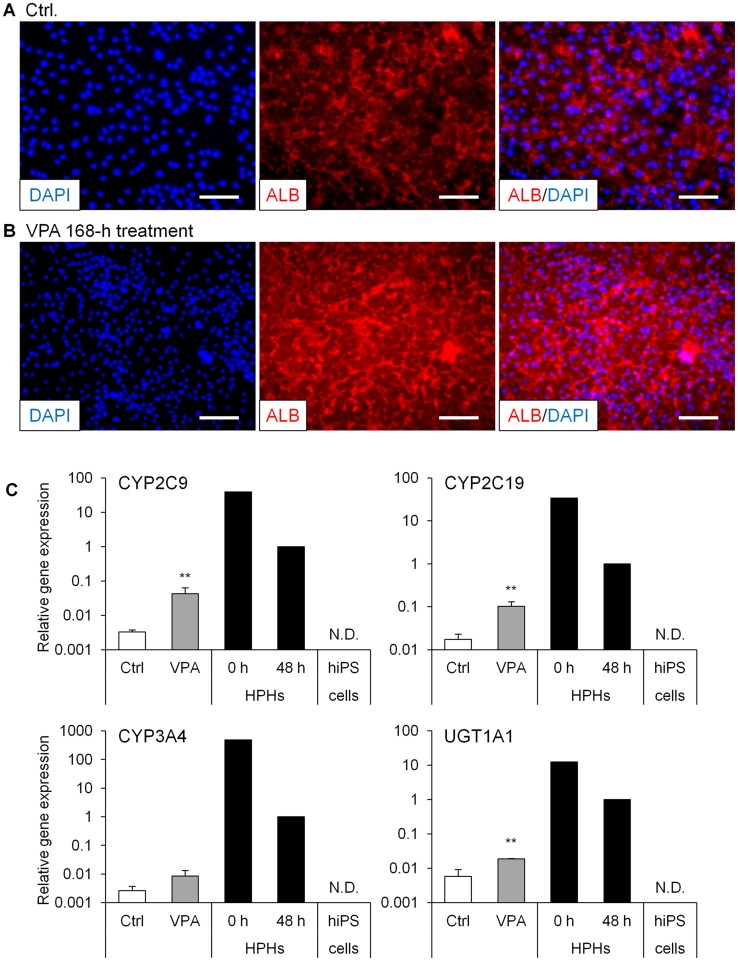
Immunofluorescence staining of ALB and effects of VPA on mRNAs encoding drug-metabolizing enzymes. Human iPS (hiPS) cells (Windy) were differentiated into hepatocytes. Valproic acid (VPA) was added to the medium for 168 h from day 12. (A, B) hiPS cell-derived hepatocyte-like cells in the absence of VPA (A) and hiPS cell-derived hepatocyte-like cells treated with VPA (B) were stained for ALB (red). Nuclei were counterstained with 4′,6-diamidino-2-phenylindole (DAPI; blue); (C) Cryopreserved human primary hepatocytes (HPHs) were cultured for 0 (just after thawing) and 48 h. Each bar represents the mean ± standard deviation (*n* = 3). The graph represents gene expression relative to that detected in human hepatocytes cultured for 48 h. Levels of statistical significance compared with VPA-untreated hepatocyte-like cells [control (Ctrl)]: **P<0.01. CYP, cytochrome P450; UGT, UDP-glucuronosyltransferase; N.D., not detected.

The morphology of hiPS cells changed dramatically during differentiation (Fig. 3). Binuclear cells, which are typical morphology of mature hepatocytes, were increased by the 168-h VPA treatment compared with the control group (Fig. 3C–F). Interestingly, vasculature-like structures in differentiated cells also appeared after the 312-h VPA treatment (data not shown). Most differentiated hiPS cells exhibited anti-ALB antibody during immunofluorescence staining after the 168-h VPA treatment, whereas the staining intensity was low in differentiated cells without the VPA treatment under the same staining conditions (Fig. 4A, B).

### Expression of drug-metabolizing enzymes in differentiated cells

**Figure 5:**
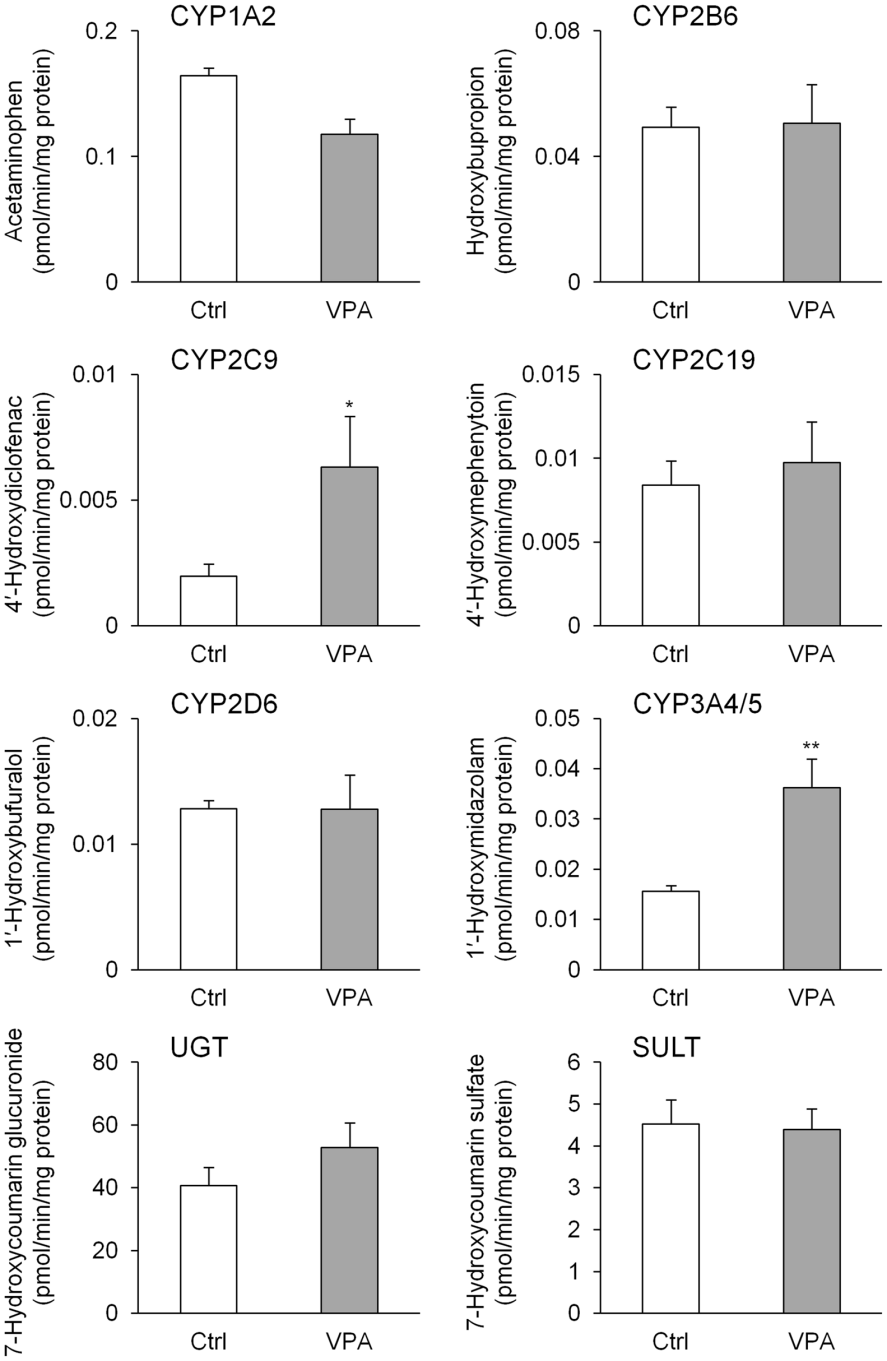
Drug-metabolizing enzyme activities in hepatocyte-like cells differentiated from hiPS cells using VPA. Human iPS (hiPS) cells (Windy) were differentiated into hepatocytes. Valproic acid (VPA) was added to medium for 168 h from day 12. Acetaminophen, hydroxybupropion, 4′-hydroxydiclofenac, 4′-hydroxymephenytoin, 1′-hydroxybufuralol, 1′-hydroxymidazolam, 7-hydroxycoumarin glucuronide, and 7-hydroxycoumarin sulfate were biotransformed from phenacetin, bupropion, diclofenac, (*S*)-mephenytoin, bufuralol, midazolam, 7-hydroxycoumarin, and 7-hydroxycoumarin by cytochrome P450 (CYP) 1A1/2, CYP2B6, CYP2C9, CYP2C19, CYP2D6, CYP3A4/5, UDP-glucuronosyltransferase (UGT), and sulfotransferase (SULT), respectively. Each bar represents the mean ± standard deviation (*n* = 3). Levels of statistical significance compared with VPA-untreated hepatocyte-like cells [control (Ctrl)]: *P<0.05 and **P<0.01.

From results of the mRNA expression analysis and immunofluorescence staining of ALB, it was suggested that the 168-h VPA treatment efficiently promoted hepatic differentiation from hiPS cell-derived HPCs. To confirm the hepatic functions of these cells, we investigated the expression of drug-metabolizing enzymes under these conditions. After 25 days of differentiation, mRNAs encoding major drug-metabolizing enzymes were detected. In particular, CYP2C9, CYP2C19, CYP3A4, and UDP-glucuronosyltransferase (UGT) 1A1 mRNAs significantly increased after the VPA treatment; in contrast, the levels of these mRNAs were low compared with those of HPHs 48 h (Fig. 4C). Furthermore, we successfully detected drug-metabolizing enzyme activities in cells that were differentiated from hiPS cells. The metabolites generated by CYP1A1/2, CYP2B6, CYP2C9, CYP2C19, CYP2D6, CYP3A4/5, UGT, and sulfotransferase was detected in the cells (Fig. 5). The CYP2C9 and CYP3A4/5 metabolites significantly increased after the 168-h VPA treatment.

### Effects of HDAC inhibitors during hepatic differentiation from hiPS cells

**Figure 6:**
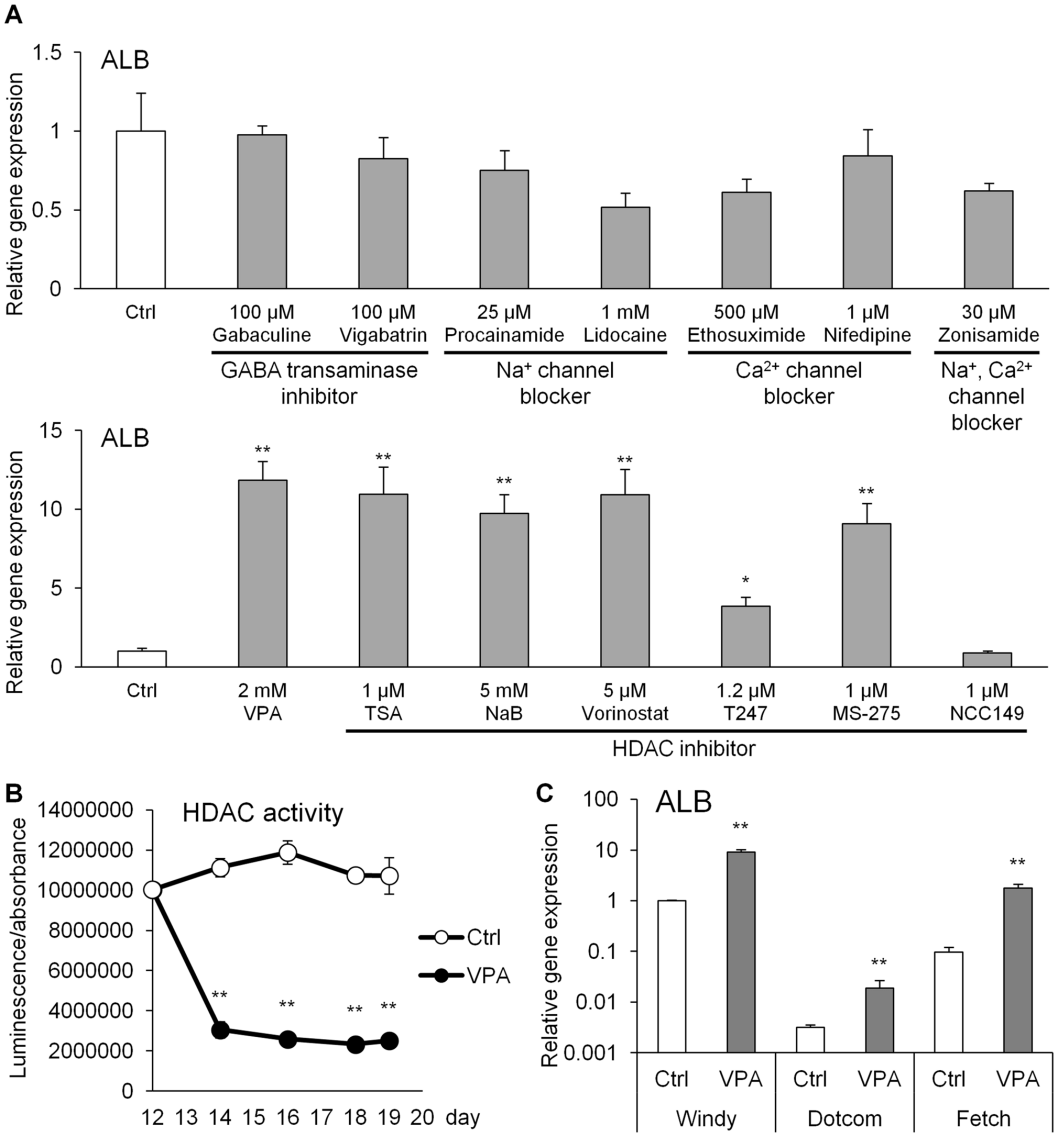
Effects of small-molecule compounds on hepatic differentiation from hiPS cells. All compounds were added to the medium for 168(A) The albumin (ALB) mRNA expression level was analyzed in hepatocyte-like cells differentiated from hiPS cells (Windy). Each bar represents the mean ± standard deviation (*n* = 3). The graph represents gene expression relative to that detected in compound-untreated hepatocyte-like cells [control (Ctrl)]. Levels of statistical significance compared with Ctrl: *P<0.05 and **P<0.01. (B) Time-dependent changes in HDAC activity in differentiating human iPS (hiPS) cells (Windy). Symbols represent the mean ± standard deviation (*n* = 4). Levels of statistical significance compared with Ctrl: **P<0.01. (C) The ALB mRNA expression level was analyzed in hepatocyte-like cells differentiated from three hiPS cell lines (Windy, Dotcom, and Fetch). Each bar represents the mean ± standard deviation (*n* = 3). The graph represents gene expression relative to that detected in VPA-untreated hepatocyte-like cells differentiated from Windy. Levels of statistical significance in each cell line compared with each Ctrl, respectively: **P<0.01. The abbreviations used are: NaB, sodium butyrate; TSA, trichostatin A.

VPA has various pharmacological actions, including the inhibition of GABA transaminase and HDAC and the blockage of ion channels. Thus, using specific inhibitors, we investigated which of these actions was involved in hepatic differentiation. ALB expression is an indicator of hepatic differentiation, because ALB is synthesized in the liver. ALB mRNA level was unaffected by treatment with GABA transaminase inhibitors and ion channel blockers for 168 h from day 12 (Fig. 6A). In contrast, treatment with all HDAC inhibitors (except NCC149) significantly increased ALB mRNA expression. Furthermore, a dramatic suppression of HDAC activity during the VPA treatment was confirmed (Fig. 6B). The ALB mRNA was expressed in multiple hiPS cell lines, and its expression after the 168-h VPA treatment was significantly higher than that detected in the VPA-untreated groups (Fig. 6C). Taken together, these data demonstrate the versatility of VPA in hepatic differentiation by acting via HDAC inhibition.

## Discussion

This study presented a new culture protocol that provides effective differentiation from hiPS cells into hepatocytes. The differentiated cells expressed hepatocyte markers and exhibited drug-metabolizing enzyme activities. These observations indicate that hiPS cells differentiated into functional hepatocyte-like cells. Furthermore, these differentiation characteristics were significantly enhanced by the administration of VPA during the final step of HPCs maturation.

Hepatic differentiation from hiPS cells exhibited differing patterns after various VPA treatment times. ALB, PXR, and TAT mRNAs increased after the 72- and 168-h VPA treatments, suggesting that VPA promotes differentiation of hiPS cell-derived HPCs into hepatocytes. However, these mRNA expression levels were decreased after the 312-h VPA treatment compared with the 168-h VPA treatment. Vasculature-like structures were observed in differentiated cells after the 312-h VPA treatment; these cells expressed the lymphatic endothelial marker gene (Fig. S1). These results suggest that VPA promotes hepatic differentiation from hiPS cell-derived HPCs in a time-dependent manner.

The inducibility of the CYP3A4 mRNA by RIF also depended on the VPA treatment times. Because RIF is a ligand of PXR, this observation may reflect PXR expression, which was low in cells that received the 312-h VPA treatments. Thus, we assumed that the 312-h VPA treatment inhibited hepatic differentiation because of a toxic effect. VPA is metabolized by CYP and the metabolites exhibit hepatotoxicity [24]. Accordingly, the 168-h VPA treatment would promote hepatic differentiation, whereas hepatotoxicity would be present after the longer VPA treatment. Thus, the duration of the VPA treatment appears to control the specificity of hepatic differentiation from hiPS cell-derived HPCs, and the present data indicate that the 168-h VPA treatment was optimal.

The liver plays a key role in drug metabolism, and it expresses phase I enzymes, such as various CYP isoforms [25], and phase II conjugating enzymes, such as UGT and sulfotransferase. Previous studies on hepatic differentiation reported that drug-metabolizing enzyme activity was determined by measuring chemiluminescence and fluorescence using P450-Glo (Promega) and ethoxyresorufin [7], [20]. A few studies measured drug-metabolizing enzyme activity using specific substrates [8]. To evaluate metabolic functions, we incubated the cells that were differentiated from hiPS cells in a medium containing substrates of drug-metabolizing enzymes. The metabolites of these substrates were detected in the supernatant after incubation; the observation that the hepatocyte-like cells generated metabolites from probe substrates was valuable. These results suggest that the present hiPS cell-derived hepatocyte-like cells have appropriate drug-metabolizing enzyme activities. VPA is known as an inhibitor of CYP2C9, CYP2C19, and CYP3A4 [26], but not as an inducer of CYPs such as RIF [27]. The activities of CYP2C9 and CYP3A4/5, however, were significantly increased by the 168-h VPA treatment. Importantly, in the present experiment, the hepatocyte-like cells used in the 168-h VPA treatment groups were cultured without VPA for the final 6 days. Accordingly, the increases in hepatic marker genes and drug-metabolizing enzyme activities observed after the 168-h VPA treatment suggest that VPA promotes hepatic differentiation.

The high reliability of ALB as a marker of hepatocyte differentiation is consistent with the fact that this protein is synthesized in the liver. In the present study, hiPS cell-derived hepatocyte-like cells expressed the ALB mRNA, which was markedly induced by the VPA treatment. In immunofluorescence experiments, ALB protein expression was also detected in almost all VPA-treated cells. In addition, the effects of VPA on ALB mRNA expression were observed in multiple differentiated hiPS cell lines, further indicating that VPA is a useful agent for generating hiPS cell-derived hepatocytes.

Previous studies demonstrated that VPA inhibits HDAC and GABA transaminase and blocks ion channels [14], [15]. Among various specific small-molecule inhibitors of HDAC and GABA transaminase and various ion channel blockers that were used during hepatic differentiation, only HDAC inhibitors functioned as effective differentiation agents. In fact, we showed that VPA inhibited HDAC activity during treatment. HDAC include various isoforms [17]. In particular, differentiation-promoting effects of the HDAC3 inhibitor T247 [22] were lower than those of other HDAC inhibitors, and the HDAC8-selective inhibitor NCC149 [23] had no effect on hepatic differentiation. In contrast, the inhibitors of HDAC1, 2, and 3, which are classified into class I HDAC [28], such as VPA, NaB, TSA, vorinostat, and MS-275, had strong effects on hepatic differentiation. Hence, the inhibition of HDAC1 and HDAC2 may promote hepatic differentiation from hiPS cell-derived HPCs. Previous studies showed that HDAC inhibitors affect DNA binding of transcriptional factors that are involved in cell growth and differentiation [14]. Although the precise mechanisms that the inhibition of HDAC promotes hepatic differentiation are unclear, the expression of genes involved in hepatic differentiation may be increased by the inhibition of HDAC in hiPS cell-derived HPCs. Ware *et al*. reported that HDAC inhibitors promote self-renewal of mouse/human ES cells, and differentiation into retinal neurons from butyrate-treated ES cells was delayed [29]. Thus, HDAC inhibitors may interfere with differentiation of ES/iPS cells. However, these HDAC inhibitors were used at low concentrations for promoting self-renewal, and at high concentrations for inducing differentiation. Moreover, they administered the inhibitors to undifferentiated ES cells. In our study, we administered VPA to HPCs. Thus, the use and purpose of HDAC inhibitors were different between our study and previous studies. We believed that VPA would have various effects depending on the cell-differentiation state or its concentration.

Dong *et al*. reported that human bone marrow stromal stem cells differentiated into hepatocyte-like cells by pretreatment with VPA [19]. In addition, undifferentiated mouse ES cells treated with VPA and without leukemia inhibitory factor, maintained the undifferentiated state, during initiation of the hepatic differentiation process [20]. Yamashita *et al*. reported that the HDAC inhibitor TSA suppressed cell growth and promoted differentiation by regulating the cell cycle in HepG2 cells (human hepatocyte carcinoma cells) [30]. Taken together, these reports suggest that HDAC inhibitory effect or cell-cycle arrest effect of VPA or TSA facilitated hepatic differentiation. However, other HDAC inhibitors remain uninvestigated. Furthermore, whether HDAC inhibitors affect the maturation process during differentiation from hiPS cells into hepatocytes remains unknown. The current findings revealed novel effects of VPA on hepatic differentiation from hiPS cells. The VPA-induced hepatic differentiation from hiPS cell-derived HPCs depended on the treatment period. The action of VPA was observed in multiple hiPS cell lines. The HDAC-inhibitory effect promoted hepatic differentiation from hiPS cells.

In conclusion, the present study demonstrated that VPA, a small-molecule compound, promoted hepatic differentiation from hiPS cells primarily by inhibiting HDAC. This new differentiation method using small-molecule compounds, which are convenient and inexpensive, would be valuable for large-scale production of functional hepatocyte-like cells differentiated from hiPS cells, because the method is simple and there is no contamination with exogenous viruses or cells. The hiPS cell-derived hepatocyte-like cells may be useful for drug development studies and liver transplantation.

## Supporting Information

Figure S1 **Effects of VPA on FLT4 expression.** Fms-related tyrosine kinase 4 (FLT4) is known to lymphatic endothelial marker. Human induced pluripotent stem cells (Windy) were differentiated into hepatocytes. Valproic acid (VPA) was added to the medium for 72 h from day 18 (72 h), 168 h from day 12 (168 h), or 312 h from day 12 (312 h) at a final concentration of 2 mM. Each bar represents the mean ± standard deviation (n = 3). The graph represents gene expression relative to that in VPA-untreated hepatocyte-like cells [control (Ctrl)].
(TIF)
